# Myopia Control Efficacy of Spectacle Lenses with Dual-Index Aspherical Lenslets: A 1-Year Randomized Clinical Trial

**DOI:** 10.1016/j.xops.2025.100766

**Published:** 2025-03-14

**Authors:** Yee Ling Wong, Andrew Tan, Ee Woon Lim, Hua Ren Chua, Liang Shen, Matthieu Guillot, Björn Drobe

**Affiliations:** 1 Research and Development Centre, EssilorLuxottica, Singapore, Singapore; 2 Bio-Statistics Unit, Yong Loo Lin School of Medicine, National University of Singapore, Singapore, Singapore; 3 Research and Development Centre, EssilorLuxottica, Paris, France

**Keywords:** Aspherical lenslets, Myopia control spectacle lenses, Optical intervention, Randomized trial

## Abstract

**Objective:**

To examine the myopia control efficacy, in terms of axial elongation, of a new myopia control spectacle lenses with Dual-Index Aspherical Lenslets (DIAL) embedded within the lens, compared with single-vision spectacle lenses (SVLs) over a 1-year period.

**Design:**

A prospective, randomized, double-masked clinical trial.

**Participants:**

Eighty children aged 8 to 13 years with myopia (spherical equivalent refraction [SER] between −0.75 and −4.75 diopters [D]) were recruited.

**Intervention:**

Participants were randomized to either the DIAL or SVL spectacle lenses group (1:1 ratio).

**Main Outcome Measures:**

Axial length (AL), noncycloplegic subjective refraction, and best-corrected visual acuity (BCVA) were measured at baseline and 6-month intervals. Questionnaires on adaptation and compliance were administered during all visits.

**Results:**

A total of 76 (N = 38 in each group) participants (mean [standard deviation] age, 10.8 [1.6] years) completed the 1-year visit. Mean (± standard error) 1-year AL change in the DIAL and SVL groups was 0.04 ± 0.02 and 0.22 ± 0.04 mm, respectively. A mean difference of −0.18 mm (95% confidence interval [CI], −0.26 to −0.10 mm; *P* < 0.001) was found. Mean 1-year SER change in the DIAL and SVL groups was −0.13 ± 0.06 and −0.39 ± 0.08 D, respectively, with a mean difference of 0.26 D (95% CI, 0.06–0.46 D; *P* = 0.01). Compared with SVL, younger children (8 to <11 years) and older children (11–13 years) in the DIAL group had significantly less axial elongation (−0.29 mm less for younger children and −0.09 mm less for older children), with greater myopia control effect of DIAL spectacle lenses among the younger group than among the older group (*P* = 0.004 for interaction). No significant differences between the lens groups were found for distance BCVA (*P* = 0.36). All participants adapted within 3 to 4 days regardless of lens group. No significant differences in mean daily wearing time were seen between the DIAL and SVL groups (*P* = 0.53).

**Conclusions:**

Dual-Index Aspherical Lenslets spectacle lenses showed good myopia control efficacy, in terms of axial elongation, compared with SVL, among children aged 8 to 13 years in Singapore.

**Financial Disclosure(s):**

Proprietary or commercial disclosures may be found in the Footnotes and Disclosures at the end of this article.

As one of the most prevalent vision disorders, myopia affects billions of people worldwide.^1^ Particularly in East Asia, the prevalence of myopia in the younger population is high,^2^ with estimated myopia prevalence in those aged 10 to 15 years ranging from 59% to 86% in Singapore, 45% to 78% in Hong Kong, and 25% to 59% in China.^3^ The myopia epidemic is a threat to global vision, as increasing levels of severity of myopia is associated with pathologies, such as myopic macular degeneration, retinal detachment, and glaucoma, which can lead to permanent blindness or visual impairment.^4^ Therefore, reducing myopia progression through myopia control strategies is vital to lowering the risk of these myopia-related pathologies.^5^

Varying myopia control efficacy has been demonstrated for an assortment of optical and pharmacological interventions for myopia control, with majority of trials investigating optical interventions.^6^ Notably, the emergence of novel spectacle lenses with lenslet-based designs has altered the landscape for optical interventions,^7^, ^8^, ^9^ offering good myopia control efficacy in terms of reducing myopia progression and axial elongation.^7^ Unexplored possibilities and underlying mechanism of spectacle lenses with lenslet-based designs remain to be discovered.

Myopia control spectacle lenses with highly aspherical lenslets (HAL) and slightly aspherical lenslets were examined in previous studies, and their myopia control efficacies in terms of reducing myopia progression and axial elongation have been demonstrated.^9^, ^10^, ^11^ Both HAL and slightly aspherical lenslets spectacle lenses consist of 11 concentric rings, each comprised of contiguous aspherical lenslets (with a diameter of 1.1 mm), positioned on the spherical front surface of the lens. In both lenses, the passage of light through these aspherical lenslets results in the creation of a 3-dimensional quantity of light in front of the retina, which is called the Volume of Myopic Defocus. Recently, we have developed a new spectacle lens, which mimics the principles of the HAL spectacle lens, namely 2 distinct signals: a correction signal focused on the retina and a second signal to control myopia progression. However, a different technology is used, which features Dual-Index Aspherical Lenslets (DIAL) embedded within the lens.

To expand on our earlier findings with the HAL spectacle lenses, we examined the myopia control efficacy, in terms of axial elongation, of the DIAL spectacle lenses compared with single-vision spectacle lenses (SVL) over a 1-year period among myopic children aged 8 to 13 years in Singapore.

## Methods

### Study Design

This study is a single, prospective, parallel, randomized, controlled, double-masked, 1-year clinical trial conducted at the Essilor Research and Development Centre Singapore, Singapore, from April 2022 to November 2023. Eligible enrolled participants are randomly assigned (1:1) to wear either DIAL spectacle lenses (treatment group) or SVL spectacle lenses (control group), based on their age, sex, and baseline right eye spherical equivalent refraction (SER) by subjective refraction, with an online software (Randola; www.rando.la).

Written informed consent was obtained from the parent or guardian. Written assent and verbal consent were obtained from the participant. Ethics Committee approval was obtained, and the study was approved by Parkway Independent Ethics Committee of the IHH Healthcare group, Singapore (Parkway Independent Ethics Committee/2021/044). The trial was registered with the Clinical Trial Registry (ClinicalTrials.gov, NCT05331378). All study procedures adhered to the tenets of the Declaration of Helsinki.

Parents or guardians, participants, and study investigators were masked to the treatment group assignment. Spectacles were labeled with participant identification but not the assigned treatment group to prevent unmasking of assigned treatment group during dispensing.

### Study Participants

Participant recruitment was conducted through several approaches, including engagement of services from an external recruitment agency (ACEllence Research), internal database, referrals, social media, and word of mouth. Telephone screening and visual screening visit were performed. Eligible participants fulfilling the inclusion and exclusion criteria were enrolled into the trial. The inclusion criteria are as follows: aged 8 to 13 years; SER by manifest refraction (noncycloplegic) between −0.75 and −4.75 diopters (D) for each eye; astigmatism not exceeding 1.50 D; anisometropia by manifest refraction not exceeding 1.00 D; and monocular best-corrected visual acuity (BCVA) of 0.10 logarithm of the minimum angle of resolution (logMAR) or better at distance for each eye. The exclusion criteria are history of myopia control treatment, any ocular pathologies, ocular surgery or systemic issues that could affect visual outcomes, and binocular vision issues (e.g., strabismus and amblyopia).

### Interventions

**Figure 1:**
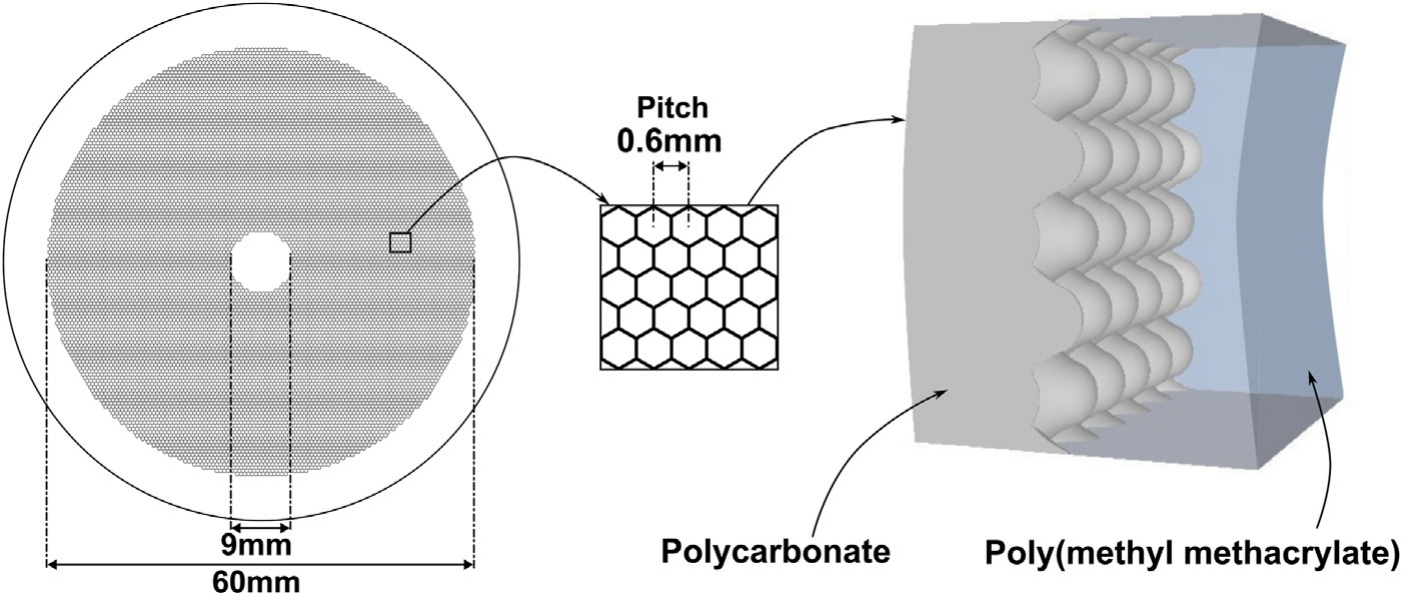
Illustration of spectacle lenses with Dual-Index Aspherical Lenslets (DIAL). On the left: frontal view of the entire lens. In the middle: cross-section incorporating elements of the array of contiguous lenslets organized in a hexagonal mesh. On the right: 3-dimensional view of the cross-section. The array of contiguous aspherical lenslets is formed at the interface of the 2 materials (polycarbonate and polymethyl methacrylate).

The DIAL spectacle lenses had a front layer made of polycarbonate (refractive index of 1.59) and a back layer made of polymethyl methacrylate (refractive index of 1.51). The array of contiguous aspherical lenslets, creating the myopia control signal, is formed at the interface between the 2 layers and arranged in a regular hexagonal mesh with a 0.6 mm pitch (i.e., center to center distance of 0.6 mm between 2 lenslets; Fig 1) in the DIAL spectacle lenses, which is different from the HAL spectacle lenses which had aspherical lenslets arranged in noncontiguous concentric rings (each ring contains contiguous lenslets).^10^ No lenslets were present in the central optical zone (9 mm), and the array of contiguous aspherical lenslets extended to a 60 mm diameter.

The array creates an average envelope parallel to the front lens surface curvature, and the aspherical lenslets geometries have been collectively calculated so that, for every gaze direction and pupil size, the image on the retina will be sharp enough to enable distance vision for the wearer. Additionally, the geometrical parameters of each aspherical lenslet have been individually calculated, so that their respective average curvature is positive relatively to the prescription, and that the combination of signals from all the lenslets generates a signal beneficial for myopia control.

Subjective refraction was used to determine the distance prescription of the spectacle lenses. The spectacle lenses were replaced at each six-month follow-up visit regardless of change in prescription.

### Outcome Variables

Ocular biometry measurement and refraction were performed every 6 months over a 1-year period. The primary outcome was change in axial length (AL) over a 1-year period. Axial length (Lenstar LS 900, Haag-Streit AG) was recorded as the mean of 3 measurements. The secondary outcome was change in SER by subjective refraction over a 1-year period. During refraction, noncycloplegic autorefraction was performed (Wave Analyzer Medica 700+ [WAM700+] wavefront aberrometer, Essilor). Subjective refraction using a phoropter (Vision R-800, Essilor) was performed, using the noncycloplegic autorefraction values as starting point, with a chart screen at 6 m. Fogging, where +1.00 D (or more) spherical lenses were added until visual acuity was worse than +0.3 logMAR, was done to reduce accommodation, as cycloplegia was not performed. Spherical equivalent refraction was calculated as sphere plus half negative cylinder.

Other outcomes include visual performance (distance and near BCVA) during dispensing visits, time needed to adapt to the lenses (no reported complaints or discomfort), and lens wearing compliance assessed via questionnaires at 6-month follow-up visits. Distance BCVA using manifest refraction with study device was measured using a chart screen (CSPOLA600, Essilor) under well-lit conditions at 6 m. Near BCVA with study device was measured using 100% contrast ETDRS near chart (Precision Vision) placed at 40 cm under well-lit conditions.

To assess adaptation to the study device, defined as wearing of the study device with no discomfort, issues, and drop in visual acuity, a telephone interview was conducted 3 to 4 days after dispensing. Lens wearing compliance was assessed via a self-responded questionnaire at the 6-month and 12-month follow-up visits. The average number of days for which the lenses were worn on weekdays and weekends in a week, and the average daily lens wearing hours on weekdays and weekends were recorded for each 6-month period. The mean daily lens wearing hours for each 6-month period was computed using the following equation: ([number of weekdays for which lenses were worn × average daily lens wearing hours on weekdays] + [number of weekends for which lenses were worn × average daily lens wearing hours on weekends])/7. The mean daily lens wearing hours over the entire 1-year period was calculated using the average of the mean daily lens wearing hours in the first and second 6-month period.

### Baseline Questionnaire

At baseline, information on demography (age, sex, and ethnicity) and medical history (age of myopia onset and parental myopia) were collected. Time spent outdoors and using digital devices (such as mobile phone, tablet, and computer) was assessed using questionnaires.

### Adverse Events

Frequency of visual symptoms (related to halos, hazy or blurred vision) with lens wear were assessed through self-reported questionnaires. Any adverse events, regardless of relatedness to spectacle lens wear, were documented.

### Sample Size Calculation

To attain 90% power and alpha level of 0.05 (2-tailed), the minimum sample size for each lens group was 29, assuming 0.13 mm (standard deviation of 0.15 mm) difference in 1-year AL change between treatment and control group.^10^ After accounting for 20% dropout rate, a sample size of 40 for each lens group would attain >90% power to detect differences in AL change between the lens groups over a 1-year period.

### Statistical Analysis

Intention-to-treat approach was adopted. Data of right eyes were selected for analysis, as high correlations for AL (*r* = 0.94, *P* < 0.001) and SER (*r* = 0.89, *P* < 0.001) between both eyes were observed. Change of outcomes was defined as the difference between baseline and corresponding follow-up measurements, and represented as mean and standard error of the mean. One-sample *t* test was used to determine if the mean change was different from zero (no change in outcomes) for each group. Chi-square test and Fisher exact test were used to test differences in proportion between groups for categorical data. Two-sample unpaired *t* test was used to test differences between groups for continuous data. Subgroup analyses were performed. SPSS statistical software version 24 (IBM Corp) was used for data analysis. Two-sided *P* values of <0.05 were considered as statistically significant.

## Results

### Study Population

**Figure 2:**
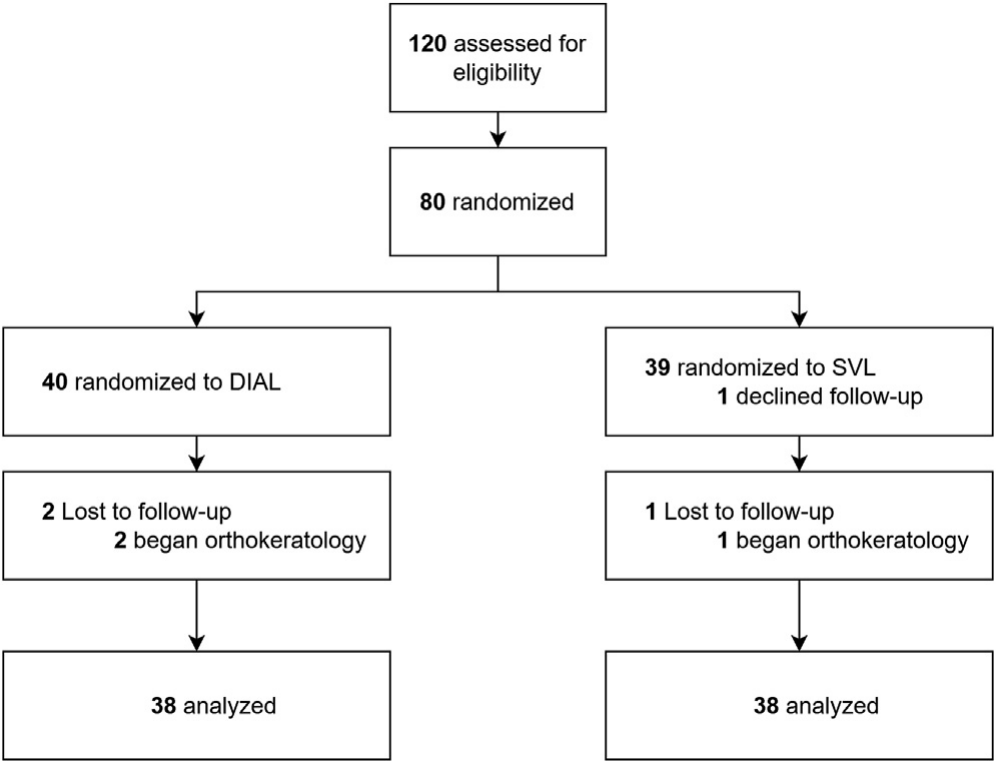
Consolidated Standards of Reporting Trials (CONSORT) flowchart. DIAL = Dual-Index Aspherical Lenslets; SVL = single-vision spectacle lenses.

A total of 80 participants aged 8 to 13 years were enrolled in the study, with equal allocation (1:1) to the DIAL and SVL groups, of which 76 (n = 38 for DIAL and n = 38 for SVL) completed the 1-year visit (Fig 2). Four children discontinued the study, with 3 reported changing to other myopia control interventions and 1 declined follow-up. Reasons for dropout were not related to study devices. The baseline characteristics of participants in both groups were similar, except for sex (*P* = 0.01; Table 1).

**Table 1:** Baseline Characteristics of Participants in Each Lens Group

Characteristics	DIAL (n = 40)	SVL (n = 40)	*P* Value
Age, yrs	10.8 ± 1.3	10.8 ± 1.8	0.93
Sex			
Male	15 (37.5)	27 (67.5)	0.01
Female	25 (62.5)	13 (32.5)	
Ethnic group			
Chinese	36 (90.0)	34 (85.0)	0.95
Malay	1 (2.5)	2 (5.0)	
Indian	1 (2.5)	2 (5.0)	
Others	2 (5.0)	2 (5.0)	
Age of myopia onset, yrs	9.1 ± 1.5	8.8 ± 1.9	0.40
Number of myopic parents			
0	5 (12.5)	2 (5.0)	0.43
1	14 (35.0)	13 (32.5)	
2	21 (52.5)	25 (62.5)	
AL, mm	24.5 ± 0.6	24.7 ± 0.8	0.22
SER by subjective refraction, D	−2.5 ± 1.0	−2.5 ± 1.1	0.71
SER by noncycloplegic autorefraction, D	−2.9 ± 1.1	−2.8 ± 1.1	0.85
Outdoor time			
Less outdoor time group (<20 hrs/wk)	22 (55.0)	16 (40.0)	0.18
More outdoor time group (≥20 hrs/wk)	18 (45.0)	24 (60.0)	
Digital device usage time			
Less digital time group (<10 hrs/wk)	18 (45.0)	19 (47.5)	0.82
More digital time group (≥10 hrs/wk)	22 (55.0)	21 (52.5)	

Data are presented as mean ± standard deviation or n (%).

AL = axial length; D = diopters; DIAL = Dual-Index Aspherical Lenslets; SER = spherical equivalent refraction; SVL = single-vision spectacle lenses.

### Primary Outcome: Change in AL

**Figure 3:**
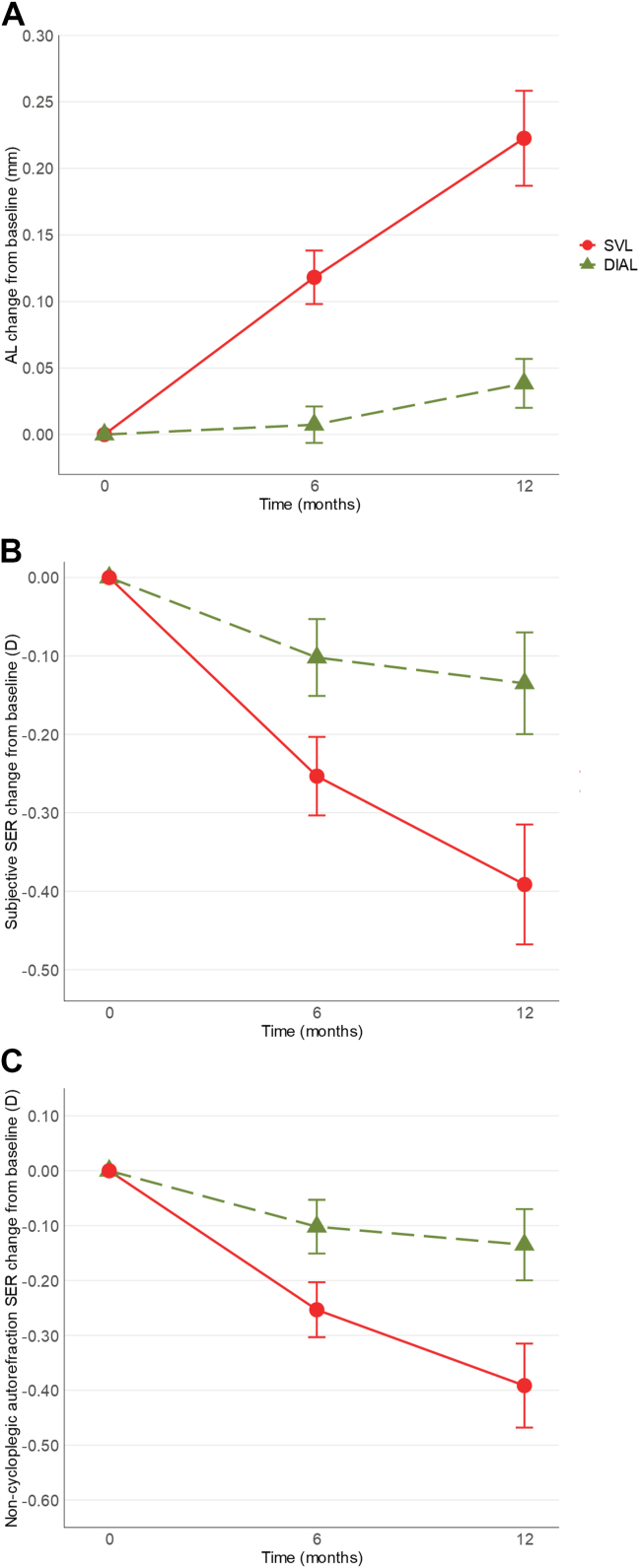
**A,** Change in unadjusted AL. **B,** Change in SER by subjective refraction. **C,** Change in SER by noncycloplegic autorefraction. Spectacle lenses with DIAL are represented in green with triangular markers, while SVL are represented in red with round markers. Error bars represent standard errors of the mean. AL = axial length; D = diopters; DIAL = Dual-Index Aspherical Lenslets; SER = spherical equivalent refraction; SVL = single-vision spectacle lenses.

Over a 1-year period, the mean (± standard error) change in AL was 0.04 ± 0.02 and 0.22 ± 0.04 mm in the DIAL and SVL groups, respectively (Fig 3A). The DIAL group had significantly less axial elongation than the SVL group (mean difference of −0.18 [95% confidence interval {CI}, −0.26 to −0.10] mm; *P* < 0.001). Significant changes in AL (compared with zero change) were found for both DIAL and SVL groups (*P* = 0.04 and *P* < 0.001, respectively).

**Figure 4:**
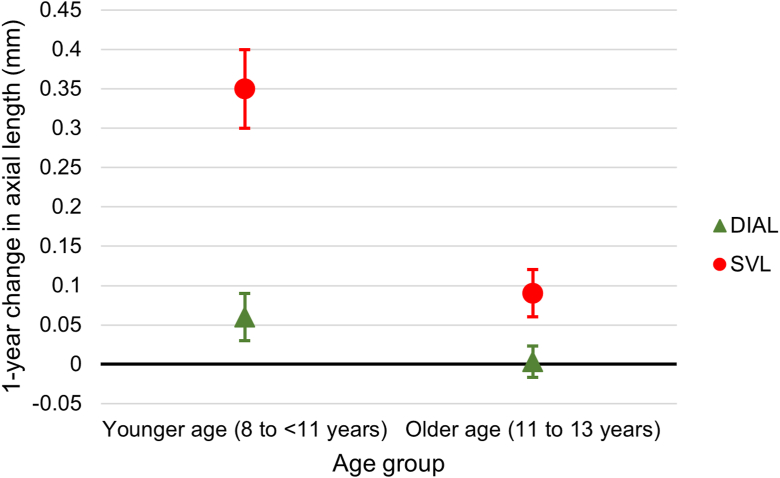
Spectacle lenses with DIAL are represented in green with triangular markers, whereas SVL are represented in red with round markers. Error bars represent standard errors of the mean. DIAL = Dual-Index Aspherical Lenslets; SVL = single-vision spectacle lenses.

As compared with participants in the control SVL group, those in the younger age group (8 to <11 years) and older age group (11–13 years) wearing DIAL spectacle lenses had significantly less axial elongation (−0.29 [95% CI, −0.41 to −0.17] mm less for younger age group [mean of 0.06 mm for DIAL versus 0.35 mm for SVL; *P* < 0.001] and −0.09 [95% CI, −0.16 to −0.01] mm less for older age group [mean of 0.00 mm for DIAL versus 0.09 mm for SVL; *P* = 0.02]; Table S2, available at www.ophthalmologyscience.org). The myopia control effect of DIAL spectacle lenses was greater among the younger age group by 0.20 mm than among the older age group (*P* = 0.004 for effect modification between age group and lens group; Fig 4). Changes in AL were significantly correlated with age in both the DIAL (*r*^2^ = −0.35; *P* = 0.03) and SVL (*r*^2^ = −0.66; *P* < 0.001) groups. In contrast, the myopia control effect of DIAL spectacle lenses was not significantly different among the group with moderate myopia and among the group with low myopia (Table S2). Likewise, the effect of DIAL spectacle lenses on axial elongation was not substantially altered by sex, ethnic group, age of myopia onset, number of myopic parents, AL, lens wearing time, outdoor time, and digital device usage time (Table S2).

### Secondary Outcome: Change in SER by Subjective Refraction

The mean 1-year change in SER by subjective refraction was −0.13 ± 0.06 and −0.39 ± 0.08 D in the DIAL and SVL groups, respectively (Fig 3B), where the DIAL group had significantly less myopia progression than the SVL group (mean difference of 0.26 [95% CI, 0.06–0.46] D; *P* = 0.01). Significant changes in SER (compared with zero change) were found for both DIAL and SVL groups (*P* = 0.04 and *P* < 0.001, respectively).

Participants with moderate myopia (SER ≤ −3 D) wearing DIAL spectacle lenses had significantly less myopia progression (0.55 [95% CI, 0.30–0.81] D less [mean of 0.10 D for DIAL versus −0.45 D for SVL; *P* < 0.001]; Table S3, available at www.ophthalmologyscience.org), compared with participants with moderate myopia wearing SVL spectacle lenses. On the contrary, participants with low myopia (−0.75 D greater than or equal to SER > −3 D) wearing DIAL spectacle lenses did not have significantly less myopia progression (0.12 [95% CI, −0.15 to 0.38] D less [mean of −0.23 D for DIAL versus −0.35 D for SVL; *P* = 0.39]). The myopia control effect of DIAL spectacle lenses was greater among the group with moderate myopia than among the group with low myopia (*P* = 0.04 for effect modification between baseline SER group and lens group). The effect of DIAL spectacle lenses on myopia progression was not substantially altered by age, sex, ethnic group, age of myopia onset, number of myopic parents, AL, lens wearing time, outdoor time, and digital device usage time (Table S3).

### Secondary Outcome: Change in SER by Noncycloplegic Autorefraction

The mean 1-year change in SER by noncycloplegic autorefraction was −0.01 ± 0.06 and −0.39 ± 0.12 D in the DIAL and SVL groups, respectively (Fig 3C), where the DIAL group had significantly less myopia progression than the SVL group (mean difference of 0.38 [95% CI, 0.10–0.65] D; *P* = 0.008). Significant change in SER (compared with zero change) was found for the SVL group (*P* = 0.003), but no significant change in SER was found for the DIAL group (*P* = 0.88).

### Visual Performance, Adaptation, and Compliance

In terms of visual performance at dispensing, no significant differences between the DIAL and SVL groups were found for distance BCVA (−0.06 ± 0.06 and −0.05 ± 0.07 logMAR, respectively; *P* = 0.36) and near BCVA (−0.11 ± 0.13 and −0.11 ± 0.12 logMAR, respectively; *P* = 0.88; Table S4, available at www.ophthalmologyscience.org). For adaptation to the study device, all participants adapted within 3 to 4 days regardless of lens group. No significant differences in mean daily wearing time were seen between the DIAL and SVL groups (12.8 ± 3.2 and 13.3 ± 3.3 hours/day, respectively; *P* = 0.53).

### Adverse Events

No severe adverse events were reported. Twelve adverse events were reported, of which most were mild and unrelated to the study device, with 2 on mild discomfort after wearing study device, 6 on mild infectious illnesses, 3 on superficial injury, and 1 on fracture.

## Discussion

In this randomized trial of myopic children aged 8 to 13 years in Singapore, DIAL spectacle lenses demonstrate good myopia control efficacy, with 0.18 mm of reduced axial elongation compared with SVL spectacle lenses after 1 year. In terms of refraction, DIAL spectacle lenses reduced myopia progression by 0.26 D compared with SVL spectacle lenses after 1 year. No intervention-related adverse event was reported. Visual acuity, adaptation, and compliance were not impacted by the lens design.

Using a different technology of embedded lenslets, the DIAL spectacle lenses mimic the design principles of the HAL spectacle lenses, and similar myopia control efficacies between these lenses were found. Comparing the cumulative absolute reduction in axial elongation (CARE),^12^ a robust metric independent of age that accounts for reduction of efficacy over time, our data showed that the CARE of the DIAL spectacle lenses at 0.18 mm was not significantly different from that of the HAL spectacle lenses at 0.21 mm after 1 year,^10^ showing comparable myopia control efficacies between the DIAL and HAL spectacle lenses. Across the CARE spectrum, CARE of the DIAL spectacle lenses is amongst the highest for myopia control treatments.^13^

Effect modification between lens group and age group for axial elongation was detected, where the effect of myopia control with DIAL spectacle lenses was significantly greater in younger children than older children. This contrasts with the findings of HAL spectacle lenses, where no significant association with age was detected.^10^ This presents DIAL spectacle lenses as a prominent myopia control intervention for younger children with myopia onset during early childhood to slow axial elongation and reduce pathologies related to excessive axial growth in later life.

Sex may have a role to play in axial elongation among myopic children.^14^ Brennan and associates found that axial elongation rate in myopes may be ≥10% higher in girls than in boys, which may be due to the age range of the study cohort and onset of puberty.^14^ Considering the higher proportion of girls in the DIAL group (62.5%) compared with the SVL group (32.5%), the mean axial elongation rate in the DIAL group may have been slightly inflated, which may in turn potentially underestimate the efficacy of the DIAL spectacle lenses.

In the SVL control group of our trial, we found mean 1-year axial elongation of 0.35 and 0.09 mm among children aged 8 to 10 and 11 to 13 years, respectively. Our observations align well with the longitudinal findings from the Singapore Cohort Study of the Risk Factors for Myopia, where axial growth estimates of 0.28 to 0.42 and 0.07 to 0.16 mm/yr for children with persistent myopia aged 8 to 10 and 11 to 12 years, respectively, were shown.^15^ As these findings from Singapore Cohort Study of the Risk Factors for Myopia may be potentially dated, new cohort studies are needed to confirm recent trends in myopia in Singapore. Compared with the SVL group in our trial in Wenzhou, China,^10^ greater 1-year axial elongation of 0.42 and 0.30 mm was seen in those aged 8 to 10 and 11 to 13 years, respectively. The smaller 1-year axial elongation (0.20 ± 0.30 mm) reported among control eyes of mainly children of Chinese descent aged 6 to 12 years in the Atropine in the Treatment of Myopia study in Singapore also suggests greater myopia progression among children in China than those in Singapore.^16^ As varying myopia progression rates are observed between populations, it highlights the need to communicate myopia control efficacy using CARE over percentage differences.^12^

Most clinical trials on evaluating myopia control efficacy of lenslet-based spectacle lenses have been limited to children in Hong Kong or China,^7^, ^8^, ^9^ thus, this current study conducted in Singapore presents myopia control efficacy of such spectacle lenses on a different population. Potential confounders, such as outdoor and digital devices usage time, which are typically not recorded in most trials, were accounted for in this study. Also, this study achieved a relatively low dropout rate (5%).

However, there are several limitations. First, cycloplegic autorefraction was not performed due to restrictions in scope of practice in Singapore, which may result in overestimation of myopia and bias in the myopia control efficacy in terms of refraction. However, the primary outcome in this study is change in AL, which has become a preferred metric,^12^ whereas change in SER is a secondary outcome. Furthermore, reduction in axial elongation reported using CARE as the main indicator of myopia control efficacy is highly recommended.^12^ Second, the follow-up period of 1 year is relatively short, but it is sufficiently long to determine the myopia control efficacy of an intervention, as the efficacy of most myopia control interventions was found to be highest in the first year.^12^ Moreover, it is not ethical to allow myopic children to continue wearing SVL once DIAL spectacle lenses were shown to be efficacious after 1 year. Third, as this study has been conducted among Singaporean children, the generalizability of the results may be limited with the differences in environmental and genetic factors, and requires further confirmation with populations in other countries. Fourth, other visual performance metrics, such as influence on contrast sensitivity, peripheral vision, and other visual outcomes, should be evaluated. Last, subjective methods, such as phone interviews and questionnaires, were adopted, which are prone to recall bias. Future studies could use wearable devices to provide objective measurements of wearing time.

Among children aged 8 to 13 years in Singapore, DIAL spectacle lenses showed good myopia control efficacy, in terms of axial elongation, compared with SVL, after 1 year. Myopia control efficacy with DIAL spectacle lenses was significantly better in younger children than older children, compared with SVL (effect modification). The absence of treatment-related adverse events indicates safety and comfort of DIAL spectacle lenses for myopia control in children.

## Supplementary Data

TableS2

TableS3

TableS4
