# Supplementary material for: Myopia Control Efficacy of Spectacle Lenses with Dual-Index Aspherical Lenslets: A 1-Year Randomized Clinical Trial
Source: Ophthalmol Sci. 2025 Mar 14;5(4):100766. doi: 10.1016/j.xops.2025.100766 (PMC12084078; doi:10.1016/j.xops.2025.100766)
Supplement: TableS2 [file mmc1.pdf]

Table S2: Subgroup analyses of 1-year change in axial length (AL) with baseline characteristics

	Mean change in AL (95% CI)			
	DIAL (n = 38)	SVL (n = 38)	p-value	p-value for interaction
Age				
8 to <11 years (n = 41)	0.06 (0.01, 0.12)	0.35 (0.25, 0.46)	<0.001	0.004
11 to 13 years (n = 35)	0.00 (-0.05, 0.05)	0.09 (0.03, 0.15)	0.02	
Gender				
Male (n = 40)	0.04 (-0.01, 0.09)	0.20 (0.12, 0.29)	0.006	0.50
Female (n = 36)	0.04 (-0.02, 0.09)	0.26 (0.11, 0.41)	0.001	
Ethnic group				
Chinese (n = 66)	0.05 (0.01, 0.09)	0.24 (0.15, 0.32)	<0.001	0.92
Non-Chinese (n = 10)	-0.05 (-0.18, 0.08)	0.15 (0.03, 0.27)	0.02	
Age of myopia onset				
Onset between 5 to 8 years (n = 37)	0.07 (0.01, 0.13)	0.30 (0.20, 0.40)	<0.001	0.19
Onset between 9 to 13 years (n = 39)	0.01 (-0.04, 0.06)	0.14 (0.04, 0.24)	0.02	
Number of myopic parents				
0 (n = 7)	0.04 (-0.10, 0.18)	-0.03 (-0.54, 0.48)	0.48	0.08
1 (n = 27)	0.03 (-0.04, 0.09)	0.15 (0.10, 0.20)	0.002	
2 (n = 42)	0.05 (-0.01, 0.11)	0.28 (0.17, 0.39)	0.001	
AL				
Shorter baseline AL group, >24.5 mm (n = 36)	0.03 (-0.03, 0.08)	0.26 (0.09, 0.43)	0.01	0.29
Longer baseline AL group, ≤24.5 mm (n = 40)	0.05 (0.01, 0.10)	0.20 (0.13, 0.27)	0.001	
SER				
Low myopia, -0.75 D ≥ SER > -3 D (n = 49)	0.04 (0.00, 0.09)	0.23 (0.11, 0.34)	0.004	0.91
Moderate myopia, SER ≤ -3 D (n = 27)	0.03 (-0.05, 0.11)	0.22 (0.13, 0.31)	0.004	
Lens wearing time*				
Full-time wearers* (n = 49)	0.03 (-0.01, 0.07)	0.25 (0.16, 0.35)	<0.001	0.19
Part-time wearers* (n = 27)	0.05 (-0.03, 0.13)	0.16 (0.04, 0.29)	0.11	
Outdoor time				
Less outdoor time group, <20 hours/week (n = 37)	0.04 (-0.01, 0.09)	0.25 (0.13, 0.37)	0.003	0.59
More outdoor time group, ≥20 hours/week (n = 39)	0.04 (-0.03, 0.10)	0.20 (0.11, 0.30)	0.004	
Digital device usage time				

Less digital time group, <10 hours/week (n = 33)	0.03 (-0.03, 0.10)	0.26 (0.17, 0.35)	<0.001	0.38
More digital time group, ≥10 hours/week (n = 43)	0.04 (-0.01, 0.09)	0.19 (0.08, 0.31)	0.02	

Abbreviations: DIAL, spectacle lenses with Dual-Index Aspherical Lenslets; SVL, single-vision spectacle lenses; AL, axial length; and SER, spherical equivalent refraction

*Full-time wearers were defined as children who reported wearing their study devices for at least 12 hours per day every day, while part-time wearers were defined as non-full-time wearers.
