# Supplementary material for: Myopia Control Efficacy of Spectacle Lenses with Dual-Index Aspherical Lenslets: A 1-Year Randomized Clinical Trial
Source: Ophthalmol Sci. 2025 Mar 14;5(4):100766. doi: 10.1016/j.xops.2025.100766 (PMC12084078; doi:10.1016/j.xops.2025.100766)
Supplement: TableS3 [file mmc2.pdf]

Table S3: Subgroup analyses of 1-year change in spherical equivalent refraction (SER) by subjective refraction with baseline characteristics

	Mean change in SER (95% CI)			
	DIAL (n = 38)	SVL (n = 38)	p-value	p-value for interaction
Age				
8 to <11 years (n = 41)	-0.16 (-0.35, 0.03)	-0.53 (-0.78, -0.29)	0.02	0.29
11 to 13 years (n = 35)	-0.09 (-0.29, 0.10)	-0.25 (-0.44, -0.06)	0.24	
Gender				
Male (n = 40)	-0.09 (-0.25, 0.07)	-0.31 (-0.49, -0.12)	0.10	0.38
Female (n = 36)	-0.16 (-0.36, 0.04)	-0.56 (-0.86, -0.26)	0.02	
Ethnic group				
Chinese (n = 66)	-0.18 (-0.32, -0.04)	-0.41 (-0.59, -0.24)	0.03	0.40
Non-Chinese (n = 10)	0.22 (-0.04, 0.47)	-0.27 (-0.64, 0.10)	0.03	
Age of myopia onset				
Onset between 5 to 8 years (n = 37)	-0.20 (-0.42, 0.02)	-0.53 (-0.75, -0.30)	0.04	0.40
Onset between 9 to 13 years (n = 39)	-0.08 (-0.26, 0.09)	-0.24 (-0.45, -0.03)	0.22	
Number of myopic parents				
0 (n = 7)	0.03 (-0.53, 0.58)	-0.13 (-1.71, 1.46)	0.68	0.13
1 (n = 27)	-0.13 (-0.33, 0.08)	-0.15 (-0.31, 0.01)	0.82	
2 (n = 42)	-0.18 (-0.39, 0.02)	-0.55 (-0.77, -0.33)	0.02	
AL				
Shorter baseline AL group, >24.5 mm (n = 36)	-0.11 (-0.31, 0.09)	-0.45 (-0.76, -0.13)	0.052	0.51
Longer baseline AL group, ≤24.5 mm (n = 40)	-0.16 (-0.34, 0.01)	-0.36 (-0.55, -0.17)	0.14	
SER				
Low myopia, -0.75 D ≥ SER > -3 D (n = 49)	-0.23 (-0.40, -0.06)	-0.35 (-0.57, -0.12)	0.39	0.04
Moderate myopia, SER ≤ -3 D (n = 27)	0.10 (-0.05, 0.25)	-0.45 (-0.68, -0.23)	<0.001	
Lens wearing time*				
Full-time wearers* (n = 49)	-0.08 (-0.24, 0.07)	-0.47 (-0.67, -0.26)	0.004	0.09
Part-time wearers* (n = 27)	-0.22 (-0.48, 0.03)	-0.25 (-0.49, -0.01)	0.87	
Outdoor time				
Less outdoor time group, <20 hours/week (n = 37)	-0.16 (-0.35, 0.03)	-0.51 (-0.77, -0.25)	0.02	0.48
More outdoor time group, ≥20 hours/week (n = 39)	-0.10 (-0.31, 0.10)	-0.31 (-0.51, -0.11)	0.15	
Digital device usage time				

Less digital time group, <10 hours/week (n = 33)	-0.15 (-0.39, 0.09)	-0.44 (-0.70, -0.18)	0.09	0.75
More digital time group, ≥10 hours/week (n = 43)	-0.13 (-0.29, 0.04)	-0.35 (-0.56, -0.15)	0.08	

Abbreviations: DIAL, spectacle lenses with Dual-Index Aspherical Lenslets; SVL, single-vision spectacle lenses; AL, axial length; and SER, spherical equivalent refraction

*Full-time wearers were defined as children who reported wearing their study devices for at least 12 hours per day every day, while part-time wearers were defined as non-full-time wearers.
