# Supplementary material for: Myopia Control Efficacy of Spectacle Lenses with Dual-Index Aspherical Lenslets: A 1-Year Randomized Clinical Trial
Source: Ophthalmol Sci. 2025 Mar 14;5(4):100766. doi: 10.1016/j.xops.2025.100766 (PMC12084078; doi:10.1016/j.xops.2025.100766)
Supplement: TableS4 [file mmc3.pdf]

Table S4: Visual performance, adaptation, and compliance

	DIAL	SVL	p-value
Distance BCVA, logMAR			
Dispensing at baseline	-0.06 ± 0.06	-0.05 ± 0.07	0.36
Dispensing at 6-month follow-up	-0.11 ± 0.06	-0.08 ± 0.06	**0.01**
Near BCVA, logMAR			
Dispensing at baseline	-0.11 ± 0.13	-0.11 ± 0.12	0.88
Dispensing at 6-month follow-up	-0.14 ± 0.10	-0.14 ± 0.09	0.99
Adaptation			
Dispensing at baseline			
Adapted	38 (100.0)	38 (100.0)	-
Not adapted	0 (0.0)	0 (0.0)	
Dispensing at 6-month follow-up			
Adapted	38 (100.0)	38 (100.0)	-
Not adapted	0 (0.0)	0 (0.0)	
Mean daily lens wearing hours, hours per day			
6-month follow-up	12.8 ± 3.3	13.2 ± 3.6	0.67
12-month follow-up	12.8 ± 3.7	13.4 ± 3.5	0.47
Over entire 1-year period	12.8 ± 3.2	13.3 ± 3.3	0.53

Data are presented as mean ± SD or n (%). Abbreviations: DIAL, spectacle lenses with Dual-Index Aspherical Lenslets; SVL, single-vision spectacle lenses; BCVA, best-corrected visual acuity; and SD, standard deviation.
